# Metagenomic Analysis of the Virome of Mosquito Excreta

**DOI:** 10.1128/mSphere.00587-20

**Published:** 2020-09-09

**Authors:** Ana L. Ramírez, Agathe M. G. Colmant, David Warrilow, Bixing Huang, Alyssa T. Pyke, Jamie L. McMahon, Dagmar B. Meyer, Rikki M. A. Graham, Amy V. Jennison, Scott A. Ritchie, Andrew F. van den Hurk

**Affiliations:** a College of Public Health, Medical and Veterinary Sciences, James Cook University, Smithfield, Queensland, Australia; b Australian Institute of Tropical Health and Medicine, Smithfield, Queensland, Australia; c School of Chemistry and Molecular Biosciences, The University of Queensland, St. Lucia, Queensland, Australia; d Australian Infectious Diseases Research Centre, The University of Queensland, St. Lucia, Queensland, Australia; e Public Health Virology, Forensic and Scientific Services, Department of Health, Coopers Plains, Queensland, Australia; f Public Health Microbiology, Forensic and Scientific Services, Department of Health, Coopers Plains, Queensland, Australia; University of Wisconsin–Madison

**Keywords:** excreta, metagenomics, mosquito, next-generation sequencing, virome

## Abstract

Traditional screening for arboviruses in mosquitoes requires *a priori* knowledge and the utilization of appropriate assays for their detection. Mosquitoes can also provide other valuable information, including unexpected or novel arboviruses, nonarboviral pathogens ingested from hosts they feed on, and their own genetic material. Metagenomic analysis using next-generation sequencing (NGS) is a rapidly advancing technology that allows us to potentially obtain all this information from a mosquito sample without any prior knowledge of virus, host, or vector. Moreover, it has been recently demonstrated that pathogens, including arboviruses and parasites, can be detected in mosquito excreta by molecular methods. In this study, we investigated whether RNA viruses could be detected in mosquito excreta by NGS. Excreta samples were collected from *Aedes vigilax* and *Culex annulirostris* experimentally exposed to either Ross River or West Nile viruses and from field mosquitoes collected across Queensland, Australia. Total RNA was extracted from the excreta samples, reverse transcribed to cDNA, and sequenced using the Illumina NextSeq 500 platform. Bioinformatic analyses from the generated reads demonstrate that mosquito excreta provide sufficient RNA for NGS, allowing the assembly of near-full-length viral genomes. We detected Australian Anopheles totivirus, Wuhan insect virus 33, and Hubei odonate virus 5 and identified seven potentially novel viruses closely related to members of the order *Picornavirales* (2/7) and to previously described, but unclassified, RNA viruses (5/7). Our results suggest that metagenomic analysis of mosquito excreta has great potential for virus discovery and for unbiased arbovirus surveillance in the near future.

**IMPORTANCE** When a mosquito feeds on a host, it ingests not only its blood meal but also an assortment of microorganisms that are present in the blood, thus acting as an environmental sampler. By using specific tests, it is possible to detect arthropod-borne viruses (arboviruses) like dengue and West Nile viruses in mosquito excreta. Here, we explored the use of next-generation sequencing (NGS) for unbiased detection of RNA viruses present in excreta from experimentally infected and field-collected mosquitoes. We have demonstrated that mosquito excreta provide a suitable template for NGS and that it is possible to recover and assemble near-full-length genomes of both arboviruses and insect-borne viruses, including potentially novel ones. These results importantly show the direct practicality of the use of mosquito excreta for NGS, which in the future could be used for virus discovery, environmental virome sampling, and arbovirus surveillance.

## Abstract (precis)

When a mosquito feeds on a host, it ingests not only its blood meal but also an assortment of microorganisms that are present in the blood, thus acting as an environmental sampler. By using specific tests, it is possible to detect arthropod-borne viruses (arboviruses) like dengue and West Nile viruses in mosquito excreta. Here, we explored the use of next-generation sequencing (NGS) for unbiased detection of RNA viruses present in excreta from experimentally infected and field-collected mosquitoes. We have demonstrated that mosquito excreta provide a suitable template for NGS and that it is possible to recover and assemble near-full-length genomes of both arboviruses and insect-borne viruses, including potentially novel ones. These results importantly show the direct practicality of the use of mosquito excreta for NGS, which in the future could be used for virus discovery, environmental virome sampling, and arbovirus surveillance.

## INTRODUCTION

In effect, female mosquitoes act as environmental samplers (“biological syringes”) that feed on the blood of a variety of vertebrate hosts (1). Mosquitoes harbor arthropod-borne viruses (arboviruses), which are capable of replicating within and being transmitted by mosquito vectors to vertebrate hosts, and insect-specific viruses (ISV) (2), as well as nonarboviruses that do not replicate in mosquitoes but might be present in hosts they feed upon (3). Traditionally, molecular assays widely utilized in arbovirus surveillance programs screen only for known and characterized endemic and enzootic viruses, including broadly reactive pan-flavivirus and pan-alphavirus assays. It is likely that many other viruses, regardless of pathogenicity, may remain undetected. Metagenomic analysis using next-generation sequencing (NGS) has proven to be useful even when a finite amount of sample is available and has allowed the unbiased identification of viruses, mosquito species, and endosymbionts, such as *Wolbachia*, from a single mosquito in a single reaction (4). Viral metagenomics is also versatile and has been successfully used in Australia for the identification of multiple arboviruses, including novel rhabdoviruses, bunyaviruses (5), and mesoniviruses (6) from field-collected mosquitoes.

Vector-enabled metagenomics could also be used as a tool to monitor human and animal diseases (1), an application often referred to as xenosurveillance (3). Xenosurveillance offers an alternative to directly sampling hosts, which involves a time-consuming process, that for humans, requires individual informed consent or, for animals, necessitates animal ethics approval prior to commencement. Alternatively, by proxy, xenosurveillance using mosquitoes has been successfully used to detect circulating H5N1 influenza virus (7), Epstein-Barr virus, canine distemper virus (3), human herpesvirus, human papillomaviruses, anelloviruses and circoviruses, among others (8). This methodology has also been used to study other pathogens, such as filarial parasites (9) or apicomplexans (10). In Sri Lanka, xenosurveillance has been successfully used to map areas with persistent circulation of Wuchereria bancrofti after mass drug administration programs (11).

Although metagenomic approaches are becoming more accessible, the costs associated with processing many samples can be prohibitive, especially in low-resource settings (12). However, analyzing mosquito excreta has the advantage of reducing the number of samples that need to be processed (1 or 2 samples versus several pools of mosquitoes in each trap). Metagenomic analysis of mosquito excreta could potentially allow unbiased identification of circulating viruses (pathogenic and nonpathogenic) from a given locale in a single reaction from a single sample.

Recently, arboviruses such as the dengue viruses (DENVs), Ross River virus (RRV), and West Nile virus (WNV) have been detected in mosquito excreta by reverse-transcription real-time PCR (RT-rtPCR) by us and others (13, 14). Additionally, hepatitis B virus, which does not replicate in the mosquito, has been detected in mosquito excreta by RT-PCR and Southern blotting up to 72 h after the ingestion of an infectious blood meal (15). A system to collect mosquito excreta in the field for the detection of circulating arboviruses has been developed, with mosquito excreta samples having been found to be positive for WNV, RRV, and Murray Valley encephalitis virus (MVEV) in Australia (16).

In this study, we evaluated the application of NGS for unbiased detection of RNA viruses, first in excreta harvested from experimentally infected mosquitoes and, later, in excreta of field-collected mosquitoes sampled from different locations in the state of Queensland, Australia. The sampling locations in Queensland have a history of transmission of medically important arboviruses, such as RRV, WNV, and Barmah Forest virus (17, 18), as well as evidence of a diverse range of insect-specific viruses (19, 20). This is the first reported study to investigate mosquito excreta as a viable and practical sample type for NGS-based metagenomics.

## RESULTS

### Laboratory studies.

**FIG 1:**
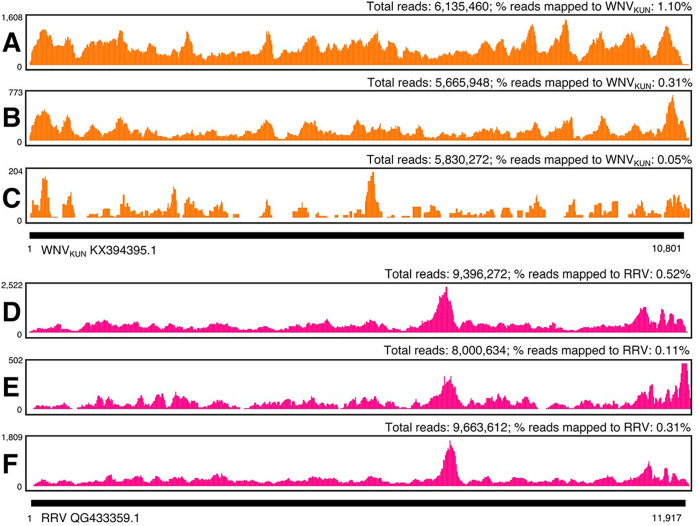
Sequence depth and coverage of virus genomes. Each sequence was obtained from excreta from a group of five experimentally infected mosquitoes exposed to either WNV_KUN_ (A to C) or RRV (D to F). Sequences were assembled to a reference sequence (GenBank accession numbers KX394395.1 and GQ433359.1 for WNV_KUN_ and RRV, respectively). Sequence depth (*y* axis) and coverage (*x* axis) are shown.

Excreta samples were collected from groups of 5 Culex annulirostris or Aedes vigilax mosquitoes exposed to either WNV (Kunjin subtype [WNV_KUN_]) or RRV, by swabbing a Parafilm disc placed in the bottom of each container (14). RNA extracted from all six excreta samples collected from groups of experimentally infected mosquitoes were positive for either WNV_KUN_ or RRV by RT-rtPCR, with mean (± standard error of the mean [SEM]) cycle threshold (*C_T_*) values of 26.3 ± 0.4 and 26.8 ± 1.0, respectively. Based on these results, six libraries were subsequently prepared and next-generation sequenced using the Illumina NextSeq platform (21). The mean (± SEM) numbers of raw reads obtained from the libraries from mosquitoes exposed to WNV_KUN_ or RRV were 5, 877,227 ± 137,555 and 9,020,173 ± 515,578, respectively. Preliminary DIAMOND/MEGAN analysis accurately classified reads obtained from experimentally infected mosquitoes as either WNV_KUN_ or RRV. Results from subsequent assembly demonstrated that excreta collected from small groups (≤5) of mosquitoes experimentally infected with arboviruses provide sufficient template for NGS, allowing the assembly of near-full-length viral genomes (Fig. 1).

### Field studies.

To determine if viral genomes could be sequenced from excreta sampled from mosquitoes collected using the methods of Meyer et al. (16), studies were conducted in Far North Queensland (FNQ) and South East Queensland (SEQ) in March and April 2018 (Fig. S1). Excreta were sampled from 19,751 mosquitoes representing at least 25 species collected in 24 traps deployed in FNQ and from 2,531 mosquitoes representing at least 18 species collected in 22 SEQ traps (Data Set S1). Viral RNA was extracted, and RRV and BFV were detected using specific RT-rtPCR assays (22, 23). RRV RNA was detected in 2 of 46 (4%) excreta samples, which had been collected from White Rock and Cattana wetlands (FNQ), with *C_T_* values of 36.9 and 36.0, respectively (Table S1). BFV RNA was detected in a single sample from Cattana wetlands (2%; *C_T_* = 36.3), which coincidentally was also positive for RRV. A total of 703 female mosquitoes from the two traps that contained the two RRV- and/or BFV-positive excreta samples mentioned above were sorted into 42 pools of ≤50 mosquitoes and screened for RRV and BFV using RT-rtPCR. Five (12%) pools were positive for RRV, and five (12%) pools were positive for BFV (Table S2).

10.1128/mSphere.00587-20.1 TABLE S1 Description of excreta samples from field-collected mosquitoes. Download Table S1, DOCX file, 0.02 MB. Copyright © 2020 Ramírez et al. 2020 Ramírez et al. This content is distributed under the terms of the Creative Commons Attribution 4.0 International license.

10.1128/mSphere.00587-20.2 TABLE S2 Mosquitoes from White Rock (FNQ8) and Cattana Wetlands (FNQ21), far North Queensland, Australia, processed for virus detection from traps that yielded excreta samples positive for Ross River and Barmah Forest viruses. Download Table S2, DOCX file, 0.02 MB. Copyright © 2020 Ramírez et al. 2020 Ramírez et al. This content is distributed under the terms of the Creative Commons Attribution 4.0 International license.

10.1128/mSphere.00587-20.4 FIG S1 Study sites in Queensland, Australia, where mosquitoes and mosquito excreta were collected. (A) Far North Queensland (FNQ); (B) South East Queensland (SEQ). Download FIG S1, EPS file, 1.0 MB. Copyright © 2020 Ramírez et al. 2020 Ramírez et al. This content is distributed under the terms of the Creative Commons Attribution 4.0 International license.

10.1128/mSphere.00587-20.5 DATA SET S1 Mosquito species identified in this study. Download Data Set S1, XLSX file, 0.02 MB. Copyright © 2020 Ramírez et al. 2020 Ramírez et al. This content is distributed under the terms of the Creative Commons Attribution 4.0 International license.

**FIG 2:**
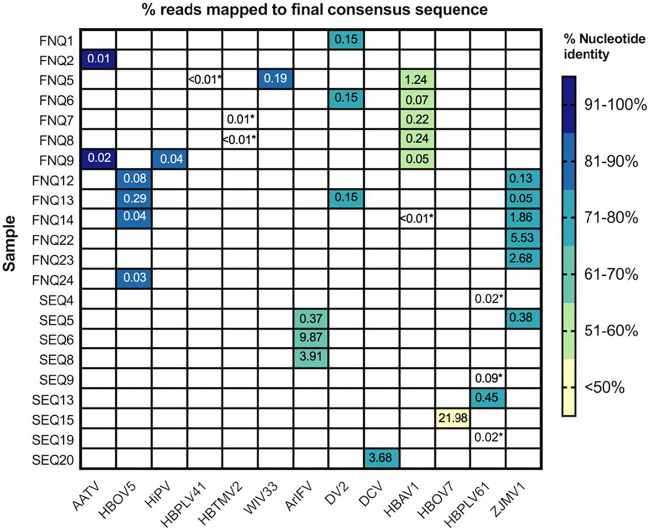
Virus genomes detected by NGS of excreta from field-collected mosquitoes. The virus names shown are from the taxonomic annotation by DIAMOND and MEGAN. These sequences were used for initial assembly and to calculate the percentage of nucleotide identity of the final consensus sequence. An asterisk indicates that only partial sequences were obtained. AATV, Australian Anopheles totivirus (NC_035674.1); HBOV5, Hubei odonate virus 5 (NC_033206.1); HiPV, Himetobi P virus (AB017037.1); HBPLV41, Hubei picorna-like virus 41 (NC_033238.1); HBTMV2, Hubei tetragnatha maxillosa virus 2 (KX883694.1); WIV33, Wuhan insect virus 33 (NC_033722.1); ArIFV, Armigeres iflavirus (LC310707.1); Daeseongdong virus 2 (NC_028489.1); DCV, Drosophila C virus (NC_001834.1); HBAV1, Hubei arthropod virus 1 (KX883297.1); HBOV7, Hubei odonate virus 7 (NC_033232.1); HBPLV61, Hubei picorna-like virus 61 (NC_033003.1); ZJMV1, Zhejiang mosquito virus 1 (NC_033716.1). Only libraries with identified virus genomes are listed.

A total of 47 libraries (including a fetal calf serum [FCS] negative control), corresponding to excreta samples collected from 11 locations, were sequenced using the Illumina NextSeq platform, with a mean (± SEM) of 12,776,515 ± 565,467 raw reads per sample (Table S1). Upon preliminary DIAMOND/MEGAN analyses using a threshold of 1,000 assigned reads, no sequences from field-collected samples were taxonomically classified as known pathogenic arboviruses, including those that were positive by RT-rtPCR for RRV and BFV. However, other RNA virus genomes were detected by NGS in 22 of the 46 excreta samples sequenced, with some samples containing up to 3 different viruses (Fig. 2).

The 13 viruses identified in this study were found to be related to members of the order *Picornavirales* and previously described unclassified RNA viruses. For three of the viruses detected (Himetobi P virus [HiPV], Hubei tetragnatha maxillosa virus 2 [HBTMV2], and Hubei picorna-like virus 41 [HBPLV41]) only partial sequences were obtained; for the rest of the viruses, we were able to assemble near-full-length genomes. Of these, Australian Anopheles totivirus (AATV), Wuhan insect virus 33 (WIV33), and Hubei odonate virus 5 (HBOV5) showed >90% amino acid (aa) identity over the RNA-dependent RNA polymerase (RdRp) to published sequences in GenBank, indicating that they correspond to strains of these viruses (19, 24). Among them, the four HBOV5 sequences were > 98% identical to each other.

**FIG 3:**
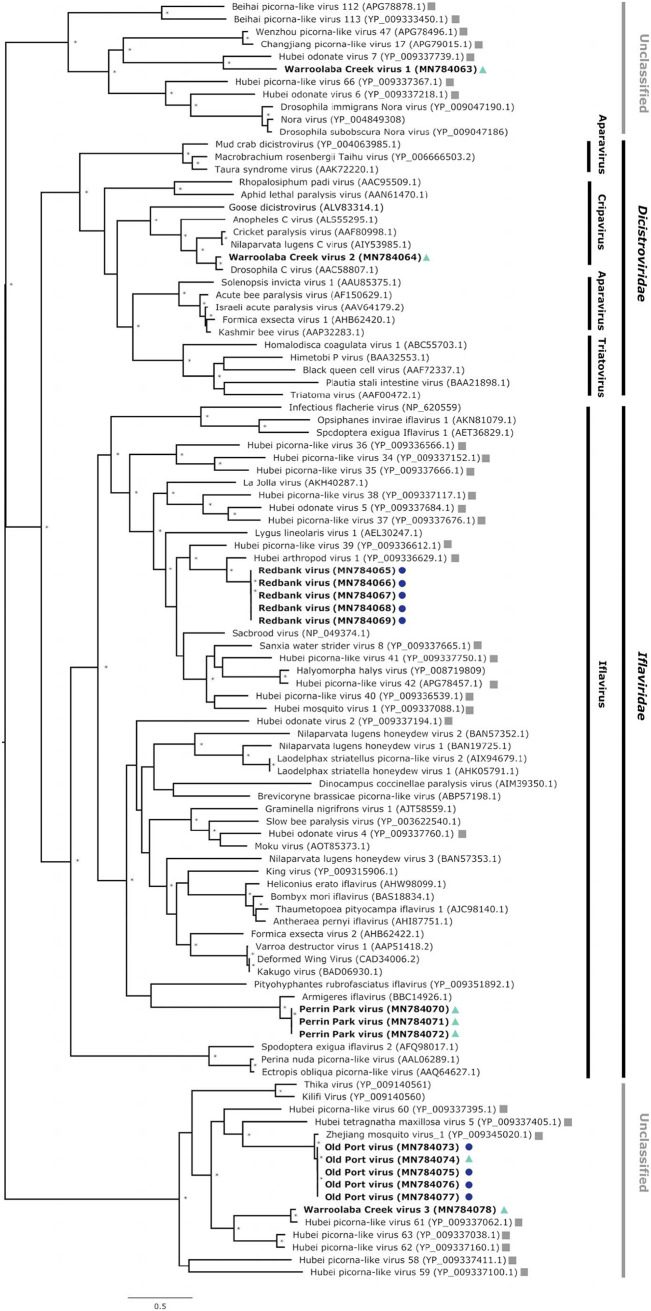
Phylogenetic relationships of viruses related to the order *Picornavirales* and other unclassified RNA viruses discovered in field mosquito excreta. A multiple-sequence alignment of the RNA-dependent RNA polymerase amino acid sequences was used to create a maximum-likelihood phylogeny using 100 bootstrap replicates; an asterisk indicates node support of >70% bootstrap support. The tree was midpoint rooted. The potential novel viruses discovered in this study are shown in bold and color coded: green triangles for samples collected in South East Queensland and blue circles for samples collected in Far North Queensland. Unclassified RNA viruses described by Shi et al. (24) are shown with gray squares. Corresponding GenBank accession numbers for compared virus sequences are provided in parentheses.

We identified seven potentially novel virus species (Table 1) and constructed phylogenetic trees based on their RdRp sequences. Warroolaba Creek virus 2 (WRCV2) is related to Drosophila C virus (DCV), a cripavirus belonging to the family *Dicistroviridae* (Fig. 3). The sequence shared ∼84% aa identity with DCV. Sequences from three samples collected in SEQ contained Perrin Park virus (PPKV), which is phylogenetically similar to Armigeres iflavirus (ArIFV; 71% aa identity), an iflavirus from the family *Iflaviridae*, which was first isolated from *Armigeres* sp. mosquitoes in the Philippines (25). The three PPKV sequences generated here were >99% identical to each other.

**TABLE 1:** Novel RNA virus sequences identified in this study

Sequence	Length (bp)	NCBI BLAST closest hit			Proposed name	Accession no.
		Closest hit	Accession no.	Identity (%)		
FNQ 1-205535.1	4,691	Daeseongdong virus 2	KU095842.1	76.4	Smithfield permutotetra-like virus	MN784079
FNQ 5-205541.1	9,499	Hubei arthropod virus 1	KX883297.1	67.2	Redbank virus	MN784066
FNQ 6-205542.1	9,722	Hubei arthropod virus 1	KX883297.1	67.2	Redbank virus	MN784067
FNQ 6-205542.2	4,594	Daeseongdong virus 2	KU095842.1	76.2	Smithfield permutotetra-like virus	MN784080
FNQ 7-205543.1	9,805	Hubei arthropod virus 1	KX883297.1	67.2	Redbank virus	MN784068
FNQ 8-205544.1	9,811	Hubei arthropod virus 1	KX883297.1	67.2	Redbank virus	MN784065
FNQ 9-205547.1	9,020	Hubei arthropod virus 1	KX883297.1	67.0	Redbank virus	MN784069
FNQ 12-205551.1	9,561	Zhejiang mosquito virus 1	KX883285.1	80.4	Old Port virus	MN784075
FNQ 13-205552.1	4,314	Daeseongdong virus 2	KU095842.1	75.9	Smithfield permutotetra-like virus	MN784081
FNQ 14-205553.1	9,562	Zhejiang mosquito virus 1	KX883285.1	80.3	Old Port virus	MN784073
FNQ 22-205608.1	9,699	Zhejiang mosquito virus 1	KX883285.1	80.3	Old Port virus	MN784077
FNQ 23-205609.1	9,644	Zhejiang mosquito virus 1	KX883285.1	80.4	Old Port virus	MN784076
SEQ 5-205641.1	8,967	Armigeres iflavirus	LC310707.1	72.0	Perrin Park virus	MN784072
SEQ 5-205641.2	9,562	Zhejiang mosquito virus 1	KX883285.1	80.3	Old Port virus	MN784074
SEQ 6-205642.1	8,985	Armigeres iflavirus	LC310707.1	72.0	Perrin Park virus	MN784071
SEQ 8-205644.1	9,117	Armigeres iflavirus	LC310707.1	72.0	Perrin Park virus	MN784070
SEQ 13-205650.1	8,513	Hubei picorna-like virus 61	KX883915.1	76.7	Warroolaba Creek virus 3	MN784078
SEQ 15-205652.1	12,762	Hubei odonate virus 7	KX883954	64.4	Warroolaba Creek virus 1	MN784063
SEQ 20-205660.1	9,304	Drosophila C virus	AF014388.1	78.6	Warroolaba Creek virus 2	MN784064

We also obtained sequences closely related to unclassified RNA viruses identified as a part of a large-scale invertebrate virosphere survey conducted on samples from China by Shi and colleagues (24) (Fig. 3). A potentially novel virus (Warroolaba Creek virus 1 [WRCV1]) from a sample from SEQ was related to Hubei odonate virus 7 (HBOV7), sharing 36% aa identity in the RdRp. Five samples from FNQ contained Redbank virus (REBV) sequences related to Hubei arthropod virus 1 (HBAV1; 59% aa identity), with >99% similarity between them. Four samples from FNQ and a single sample from SEQ contained Old Port virus (OPTV) sequences related to Zhejiang mosquito virus 1 (ZJMV1; 85% aa identity) with >97% similarity with each other. Sequences from one sample collected (Warroolaba Creek virus 3 [WRCV3]) in SEQ were phylogenetically similar to Hubei picorna-like virus 61 (HBPLV61; 83% aa similarity), which had been previously identified in mosquitoes.

**FIG 4:**
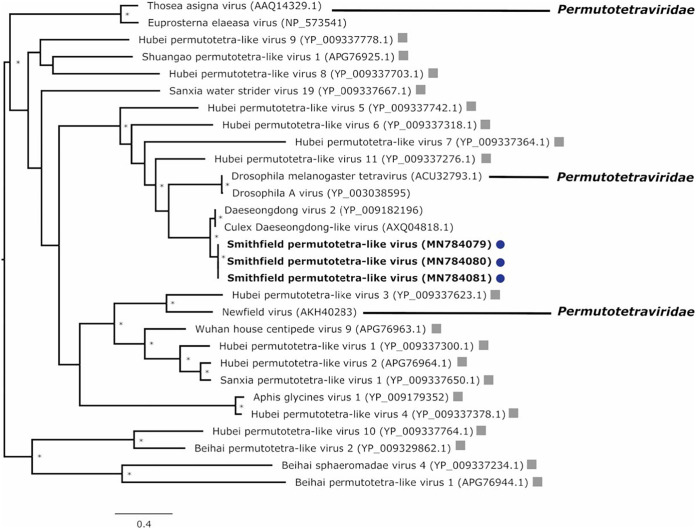
Phylogenetic relationships of unclassified RNA viruses discovered in field mosquito excreta. A multiple-sequence alignment of the RNA-dependent RNA polymerase amino acid sequences was used to create a maximum-likelihood phylogeny using 100 bootstrap replicates; an asterisk indicates node support of >70% bootstrap support. The tree was midpoint rooted. The potential novel viruses discovered in this study are shown in bold and color coded with blue circles for samples collected in Far North Queensland. Unclassified RNA viruses described by Shi et al. (24) are shown with gray squares. Corresponding GenBank accession numbers for compared virus sequences are provided in parentheses.

Finally, three samples from FNQ contained Smithfield permutotetra-like virus (SmPLV) sequences related to both Culex Daeseongdong-like virus and Daeseongdong virus 2 (DV2) (Fig. 4). The sequences shared ∼84% aa identity with both unclassified RNA viruses, which are themselves highly similar to each other (>99%) and were identified in *Culex* mosquitoes from Korea and California, respectively (26, 27). Our three SmPLV sequences were >99% identical to each other.

The FNQ collections appeared to have greater virus diversity, with full-length sequences from 6 different viruses identified only from FNQ (AATV, HBO5V, HiPV, WIV33, SmPLV, and REBV), compared with 4 identified only from SEQ (PPKV, WRCV1, WRCV2, and WRCV3). Only OPTV was detected in both FNQ and SEQ samples.

### Evaluation of rRNA in excreta samples.

Since an rRNA depletion kit was not used in our study, we evaluated the extent of rRNA presence in excreta samples where a virus was found by NGS in both laboratory-infected and field-collected mosquitoes. We did a BLAST search of a subset of reads (*n* = 1,000) against SILVA small-subunit (SSU) and large-subunit (LSU) rRNA sequences databases (28). The amount of SSU RNA and LSU rRNA varied from 12% to 75% of reads and from 19% to 93% of reads, respectively, suggesting a large amount of rRNA present in the samples (Table S3).

10.1128/mSphere.00587-20.3 TABLE S3 rRNA sequences in mosquito excreta samples determined by BLAST against SILVA SSU and LSU databases. Download Table S3, DOCX file, 0.02 MB. Copyright © 2020 Ramírez et al. 2020 Ramírez et al. This content is distributed under the terms of the Creative Commons Attribution 4.0 International license.

## DISCUSSION

Over the past decade, unbiased metagenomic analysis using NGS has become a valuable tool and revolutionized virus discovery and surveillance (29, 30). However, as with all detection-based technologies, these techniques are prone to limitations associated with sample type, integrity, availability, and amount. The current study aimed to circumvent some of these issues pertinent in surveillance strategies by coupling the less biased sampling afforded by mosquito feeding behaviors, which is further simplified by collection of excreta, with unbiased virome detection made possible via NGS. Our laboratory findings demonstrate that excreta from experimentally infected mosquitoes provide a sufficient template for sequencing and assembly of near-full-length virus genomes. However, we were unsuccessful at sequencing RRV or BFV from the two field-collected samples that were positive by RT-rtPCR. A possible explanation for this is the likelihood that NGS is not as sensitive as RT-rtPCR for detection of viruses with low titers (31) and those present in complex samples (32). In our study, samples from experimentally infected mosquitoes contained a larger amount of starting template (as evidenced by lower *C_T_* values) and were likely subjected to less sample degradation than field samples. Since the viral titers to which mosquitoes were exposed to during laboratory infection may have been higher than those encountered by mosquitoes in the wild, future investigations should include determination of the limit of detection for this method by exposing mosquitoes to different viral titers.

Despite this, we could detect arbovirus sequences in samples with *C_T_* values of ≤28.5. Among the libraries that were positive by RT-rtPCR for RRV and/or BFV but negative by NGS, all had *C_T_* values of ≥36.0, suggesting that the failure to detect these viruses may have resulted from low abundance of viral sequences. Further, our results indicate a large amount of rRNA present in mosquito excreta, with an average of more than 50% of sequence reads matching both SSU and LSU rRNA, which could potentially impact the sensitivity of virus detection. In the future, it is likely that by adding an rRNA depletion step, sensitivity can be increased. Additionally, viral enrichment protocols such as NetoVIR (33), which have been used for single-mosquito viral metagenomics (34), could also be used to increase the sensitivity for virome analysis. However, it is important to note that we were able to assemble near-full-length genomes from excreta samples where up to 95% of sequence reads corresponded to rRNA. With increased application of NGS, it is likely that new, improved protocols will be devised, increasing the efficiency and sensitivity of this sequencing platform. Coinciding with NGS technological advancements, mosquito excreta could be utilized as a valuable alternative sample for routine arbovirus surveillance, enabling the unbiased detection of arboviruses of public health importance from a single sample, thereby increasing the surveillance scope. Furthermore, this approach together with NGS metagenomic analysis of mosquito saliva deposited on honey-soaked FTA cards (35) could potentially provide a wider and more comprehensive overview of pathogenic and nonpathogenic microbiota circulating in given locales.

By performing sequencing of field-collected mosquito excreta samples, we were able to show evidence of the circulation of 13 insect-borne viruses, of which one (AATV) had been previously identified in mosquitoes and four (PPKV, SmPLV, WRCV3, and OPTV) were related to viruses previously identified in mosquitoes. Some of these viruses have been isolated and/or characterized. In the case of AATV, although the virus has not been isolated in C6/36 mosquito cells, evidence of its replication in mosquitoes has been observed by small-RNA analysis (19). ArIFV, to which PPKV is related, causes pronounced cytopathic effect in C6/C6 cells; however, its pathogenicity in mosquitoes still needs to be evaluated (25). Some iflaviruses, such as slow bee paralysis virus and infectious flacherie virus, can cause lethal infections in their host (36, 37). Viruses related to SmPLV, WRCV3, and OPTV that have been previously identified in mosquitoes remain unclassified and have been characterized only *in silico* (24, 26, 27). Clearly, more work needs to be done on the novel viruses identified in this study with respect to their phenotypic characterization.

Although these insect-borne viruses are unlikely to be associated with disease in vertebrates, they can potentially affect the vector competence of mosquitoes for pathogenic viruses, as demonstrated previously for a number of insect-specific viruses (38, 39). Because of this, elucidating the mosquito virome is critical for understanding the role a mosquito species plays in arbovirus transmission cycles and potential control strategies. Further, novel insect-specific viruses have been shown to be potentially useful as biological control agents or as platforms for vaccine and diagnostic development (2), and therefore, expanded detection of these agents could have important downstream applications.

As of 2020, the costs associated with NGS and the time and bioinformatics skills required to analyze the results from thousands to tens of thousands of mosquitoes can be prohibitive, especially in low-resource settings (12). We have shown that mosquito excreta can be used as a preliminary sample for virus discovery in field populations of mosquitoes. Using mosquito excreta has the advantage of reducing costs by requiring sequencing of only one sample from a trap, instead of multiple pools of mosquitoes, which in traps containing as many as 10,000 mosquitoes could result in 200 to 400 pools depending on pool size. Based on the results obtained from excreta, the mosquitoes could be used for subsequent sequencing or in attempts to detect virus for subsequent isolation. Indeed, we detected RRV and BFV by RT-rtPCR in mosquito pools prepared from traps that yielded RT-rtPCR-positive excreta. Although these viruses were detected using RT-rtPCR, this result highlights the utility of the excreta sampling approach in identifying viral RNA in the first instance, which, in the future, could more suitably be applied to NGS, as the sensitivity of NGS increases.

A limitation of our field study is that it is almost impossible to attribute the excreta deposited on the polycarbonate substrate to a particular insect. Although mosquitoes comprise the majority of the collections, traps used to capture mosquitoes also attract nontarget insects (40), which could feed on the honey and excrete on the substrate. This can be reflected by the detection of HBOV5, which is associated with dragonflies and damselflies, and HBTMV2, which is associated with spiders (24). To overcome this technical limitation, the mosquitoes could first be sorted and transferred to clean containers in the laboratory from which excreta would be obtained and sequenced to confirm the origin of each virus. Additionally, based on results obtained from sequencing mosquito excreta, virus detection and subsequent isolation could be attempted from the mosquito homogenates for viruses that can be grown *in vitro*. Use of traps that are highly selective for mosquitoes, such as CO_2_-baited passive box traps that do not deploy lights that attract other insects, is recommended (16, 41).

It is evident that NGS technologies have many applications for the study of vectors and the pathogens they transmit (42). Here, we have demonstrated that metagenomic analysis of mosquito excreta can be used in the context of arbovirus surveillance for virus discovery, as well as for unbiased virome sampling, particularly as costs decrease and technologies become more accessible.

## MATERIALS AND METHODS

### Excreta collection from experimentally infected mosquitoes.

Field-collected *C. annulirostris* and laboratory-reared *A. vigilax* mosquitoes were exposed to defibrinated sheep blood (Institute of Medical and Veterinary Science, Adelaide, Australia) containing either WNV (Kunjin subtype [WNV_KUN_]; 10^7.3±0.3^ 50% tissue culture infective doses [TCID_50_]/ml) or RRV (10^8.1±0.1^ TCID_50_/ml) via the hanging drop method (43) and by using a Hemotek membrane feeder (Discovery Workshops, Accrington, Lancashire, UK), respectively. For each virus, three groups of five fully engorged females were placed in 200-ml polypropylene containers modified with an insect screen floor for excreta collection (14). Four to 7 days after mosquitoes fed on the infectious blood meal, a single excreta sample was collected from each container using a cotton swab moistened with growth medium (GM) (Opti-MEM [Gibco, Invitrogen Corporation, Grand Island, NY] supplemented with 3% fetal bovine serum [In Vitro Technologies, Nobel Park North, VIC, Australia], antibiotics and antimycotics) as described by Ramírez et al. (14). The swab was then placed in a 2-ml free-standing tube containing 1 ml GM and stored at −80°C. All mosquito exposures were conducted under biosafety level 2 (BSL-2) conditions, and mosquitoes were maintained at 28°C, 75% relative humidity (RH), and a 12:12 (light:dark) photoperiod within an environmental growth cabinet.

### Excreta collection from field mosquitoes.

Mosquito excreta samples were collected in March and April 2018 in SEQ and FNQ, Australia, from different sites that encompassed a variety of vertebrate host species, including flying foxes, wading birds, livestock, and macropods (Fig. S1). Adult mosquitoes were collected using Centers for Disease Control and Prevention (CDC) model 512 light traps (John W. Hock Company, Gainesville, FL) fitted with collection containers modified from the design described in reference 16. Collection containers housed two filter paper cards soaked with blue-dyed honey and a removable polycarbonate substrate for excreta collection. All traps were baited with 1 kg of dry ice as a source of CO_2_; traps deployed in FNQ were also supplemented with 1-octen-3-ol to increase capture of mosquitoes (44). Traps were operated for 14 h overnight, before being transported to the laboratory and placed in a humidified box, where mosquitoes were allowed to feed on the honey-soaked filter paper cards for an additional 24 h to increase the amount of excreta produced. Excreta were collected from the polycarbonate insert using a moistened swab as described above and stored at −80°C. The mosquitoes from each trap were cold-anesthetized and placed in 70-ml vials before being stored at −80°C. To avoid cross-contamination, the traps and polycarbonate inserts were handled with gloves during trap assembly and retrieval, and gloves were changed after each sample was collected. After collection, the polycarbonate inserts were soaked in 1% bleach and then rinsed and wiped with 70% ethanol, while the pots and mesh inserts were wiped with 1% bleach followed by 70% ethanol.

### Virus assays.

Thawed excreta samples were shaken to release the material from the swab using a TissueLyser II (Qiagen, Hilden, Germany) for 3 min at 26 Hz and centrifuged at 14,000 × *g* for 30 s to remove droplets from the lid (14). RNA from excreta from experimentally infected mosquitoes was extracted from the supernatant using the QIAamp viral RNA minikit (Qiagen, Hilden, Germany); RNA from excreta from field-collected mosquitoes was extracted using the One-For-All nucleic acid kit (Qiagen, Hilden, Germany). Extractions were carried out with and without carrier (to avoid interference in downstream NGS), according to the manufacturer’s instructions. RNA extracted with carrier was immediately tested by RT-rtPCR, and RNA extracted without carrier was stored at −80°C for sequencing.

Excreta samples from experimentally infected mosquitoes were screened for either WNV_KUN_ (23) or RRV (22) by RT-rtPCR. Field-collected excreta samples were tested for RRV and BFV (23), as these two alphaviruses are endemic in the sampling locations. In addition, mosquitoes from traps where arboviral RNA was detected in excreta by RT-rtPCR were identified morphologically by an experienced medical entomologist (A.F.V.D.H) and placed into pools of ≤50 mosquitoes by species before being homogenized in 2 ml of GM as described above; viral RNA was extracted using the QIAamp viral RNA minikit with carrier following the manufacturer’s recommendations. Viral RNA was detected in excreta samples and mosquito pools using RT-rtPCR assays specific for RRV, WNV_KUN_, and BFV on a Rotor-Gene 600 real-time thermocycler (Qiagen, Australia). Every run included synthetic primer and probe controls, a positive extraction control (bovine viral diarrheal virus [BVDV]), a negative extraction control, and a no-template control (molecular-grade water). The results were evaluated qualitatively: for any sample, a threshold cycle number (*C_T_*) of >40 indicated that no RNA was detected (45).

### Sequencing.

Genomic DNA (gDNA) was removed from total RNA aliquots extracted without carrier RNA using the Heat & Run gDNA removal kit (ArcticZymes, Tromso, Norway). RNA was reverse transcribed, and first-strand cDNA was synthesized using the NEB Protoscript II first-strand cDNA synthesis kit (New England BioLabs, Ipswich MA) according to the manufacturer’s instructions, followed by second-strand cDNA synthesis using NEB second-strand synthesis enzyme buffer (New England BioLabs, Ipswich, MA) and second-strand DNA enzymes (DNA polymerase I [Escherichia coli], 10 U; RNase H, 0.35 U; and E. coli DNA ligase, 1.25 U; New England BioLabs, Ipswich, MA). The newly synthesized DNA was purified by ethanol precipitation. DNA libraries were prepared using the Nextera XT DNA library preparation kit (Illumina) and Illumina Nextera XT index kit. The resulting libraries were analyzed, and DNA sizing and quantification were performed using a 2200 TapeStation (Agilent Technologies). A fetal calf serum (FCS) library was prepared as described above from FCS RNA as a negative control. Libraries were diluted to 1 nM, pooled, denatured and diluted to a final concentration of 1.2 pM. Paired-end sequencing was performed using the NextSeq platform (Illumina) using a NextSeq 500 Mid Output V2 kit (Illumina) (21).

### Sequence analysis and phylogenetics.

Sequence reads were demultiplexed and adapters were removed using bcl2fastq version 2.20 (http://sapac.support.illumina.com/downloads/bcl2fastq-conversion-software-v2-20.html). An initial search of the raw sequences was conducted using DIAMOND (BLASTx) (46) against the NCBI-nr viral protein reference sequence database (downloaded on 20 March 2019). Taxonomic binning of the reads was performed using MEGAN CE version 6.15.2 (47) using the naive LCA algorithm (minimum score = 75.0; maximum expected = 0.1; top percent = 10.0; minimum support = 10). Based on these results, RNA virus sequences with at least 1,000 reads assigned as close relatives were selected as references for assembly. Viral sequences were assembled using Geneious Prime version 2019.0.4 by either *de novo* assembly or manually mapping the reads to a reference sequence obtained from GenBank. For *de novo* assembly, low-quality (Q < 30) and short (<100-nt) reads were trimmed using BBDuk before being assembled using SPAdes (48) with default parameters. Assembled sets of overlapping DNA sequences (contigs) were then mapped to a reference sequence, or the longest contigs containing an open reading frame were compared against the NCBI-nr database using BLAST (49) and used as a reference sequence for further assembly. Alternatively, raw reads were mapped against a reference sequence using default settings, and the consensus sequence of this assembly was used for subsequent assembly (19). This process was repeated until the length of the consensus sequence did not increase anymore. Reads from experimentally infected mosquitoes or from field-collected mosquitoes with a positive arbovirus RT-rtPCR result were mapped against a reference sequence.

Assembled sequences with > 90% amino acid identity with existing viruses over the RNA-dependent RNA polymerase (RdRp) were considered strains of these viruses and assumed to phylogenetically group with them. Because of this, only complete sequences with less than 90% amino acid identity to the reference sequence were included in phylogenetic analyses. The translated contigs were aligned with protein sequences obtained from GenBank using the results from BLAST and previously published phylogenetic trees of the related viruses (24, 25). Multiple protein alignments were done using MAFFT v7.388 (50) and trimmed using TrimAl (51). The optimal evolutionary model was selected using the Akaike information criterion in SMS (52). Maximum-likelihood phylogenies were generated using the Le Gascuel (LG) amino acid substitution model with 100 bootstrap replicates using PhyML v 3.3 (53).

### Evaluation of rRNA contamination in excreta samples.

Since an rRNA depletion kit was not used in our study, we evaluated the extent of rRNA contamination in our excreta samples. For this, we estimated the amount of rRNA on a subset of reads (*n* = 1,000) from all the excreta samples shown to contain a virus by NGS (from both laboratory-infected and field-collected mosquitoes) by performing a BLAST search (49) against SILVA small subunit (SSU) and large subunit (LSU) rRNA sequences databases (https://www.arb-silva.de/) (28) using Geneious Prime version 2020.2.2. Finally, for each sample, the percentage of sequences that got a hit was calculated for each database.

### Data availability.

Raw sequence reads generated from this study have been deposited in the NCBI Sequence Read Archive (SRA) repository under project number PRJNA631724. All virus genome sequences generated in this study have been deposited in GenBank under accession numbers MN784056 to MN784081.
