# Supplementary material for: Metagenomic Analysis of the Virome of Mosquito Excreta
Source: mSphere. 2020 Sep 9;5(5):e00587-20. doi: 10.1128/mSphere.00587-20 (PMC7485684; doi:10.1128/mSphere.00587-20)
Supplement: TABLE S1 [file mSphere.00587-20-st001.docx]

**Table S1.** Description of excreta samples from field-collected mosquitoes

| **Sample** | **Region** | **Location** | **Collection date** | **RT-rt PCR (C*_t_* value)** | | **Total reads** |
| --- | --- | --- | --- | --- | --- | --- |
|  |  |  |  | **RRV** | **BFV** |  |
| FNQ1 | Far North Queensland | Smithfield | 09-Apr-18 |  |  | 12,849,836 |
| FNQ2 | Far North Queensland | Smithfield | 09-Apr-18 |  |  | 16,323,204 |
| FNQ3 | Far North Queensland | Smithfield | 09-Apr-18 |  |  | 9,977,834 |
| FNQ4 | Far North Queensland | Trinity Beach | 09-Apr-18 |  |  | 12,677,268 |
| FNQ5 | Far North Queensland | Packers Camp | 11-Apr-18 |  |  | 15,409,794 |
| FNQ6 | Far North Queensland | Packers Camp | 11-Apr-18 |  |  | 8,743,892 |
| FNQ7 | Far North Queensland | White Rock | 11-Apr-18 |  |  | 11,742,104 |
| FNQ8 | Far North Queensland | White Rock | 11-Apr-18 | 36.9 |  | 15,236,836 |
| FNQ9 | Far North Queensland | Smithfield | 12-Apr-18 |  |  | 13,616,396 |
| FNQ10 | Far North Queensland | Smithfield | 12-Apr-18 |  |  | 12,907,916 |
| FNQ11 | Far North Queensland | Smithfield | 12-Apr-18 |  |  | 12,724,850 |
| FNQ12 | Far North Queensland | Port Douglas | 13-Apr-18 |  |  | 14,794,748 |
| FNQ13 | Far North Queensland | Port Douglas | 13-Apr-18 |  |  | 13,773,766 |
| FNQ14 | Far North Queensland | Port Douglas | 13-Apr-18 |  |  | 13,650,320 |
| FNQ15 | Far North Queensland | White Rock | 16-Apr-18 |  |  | 10,906,464 |
| FNQ16 | Far North Queensland | White Rock | 16-Apr-18 |  |  | 12,146,108 |
| FNQ17 | Far North Queensland | Central swamp | 16-Apr-18 |  |  | 25,046,584 |
| FNQ18 | Far North Queensland | Central swamp | 16-Apr-18 |  |  | 11,113,836 |
| FNQ19 | Far North Queensland | Packers Camp | 17-Apr-18 |  |  | 26,519,388 |
| FNQ20 | Far North Queensland | Packers Camp | 17-Apr-18 |  |  | 20,409,440 |
| FNQ21 | Far North Queensland | Cattana Wetlands | 17-Apr-18 | 36.0 | 36.3 | 17,320,194 |
| FNQ22 | Far North Queensland | Port Douglas | 18-Apr-18 |  |  | 12,570,618 |
| FNQ23 | Far North Queensland | Port Douglas | 18-Apr-18 |  |  | 12,121,104 |
| FNQ24 | Far North Queensland | Port Douglas | 18-Apr-18 |  |  | 15,378,002 |
| SEQ1 | South East Queensland | Warrill View | 07-Mar-18 |  |  | 12,454,200 |
| SEQ2 | South East Queensland | Warrill View | 07-Mar-18 |  |  | 11,719,252 |
| SEQ3 | South East Queensland | Warrill View | 07-Mar-18 |  |  | 14,622,684 |
| SEQ4 | South East Queensland | Warrill View | 07-Mar-18 |  |  | 13,828,138 |
| SEQ5 | South East Queensland | Toowong | 12-Mar-18 |  |  | 11,303,708 |
| SEQ6 | South East Queensland | Toowong | 12-Mar-18 |  |  | 8,748,950 |
| SEQ7 | South East Queensland | Warrill View | 14-Mar-18 |  |  | 10,118,292 |
| SEQ8 | South East Queensland | Warrill View | 14-Mar-18 |  |  | 11,277,978 |
| SEQ9 | South East Queensland | Warrill View | 14-Mar-18 |  |  | 7,115,728 |
| SEQ10 | South East Queensland | Warrill View | 14-Mar-18 |  |  | 12,809,820 |
| SEQ11 | South East Queensland | Toowong | 19-Mar-18 |  |  | 5,416,744 |
| SEQ12 | South East Queensland | Warrill View | 20-Mar-18 |  |  | 11,430,744 |
| SEQ13 | South East Queensland | Warrill View | 20-Mar-18 |  |  | 9,523,870 |
| SEQ14 | South East Queensland | Warrill View | 20-Mar-18 |  |  | 9,050,850 |
| SEQ15 | South East Queensland | Warrill View | 20-Mar-18 |  |  | 11,727,168 |
| SEQ16 | South East Queensland | Hemmant 2 | 26-Mar-18 |  |  | 11,476,134 |
| SEQ17 | South East Queensland | Hemmant 2 | 26-Mar-18 |  |  | 7,325,404 |
| SEQ18 | South East Queensland | Coopers Plains | 26-Mar-18 |  |  | 11,402,318 |
| SEQ19 | South East Queensland | Warrill View | 27-Mar-18 |  |  | 12,517,160 |
| SEQ20 | South East Queensland | Warrill View | 27-Mar-18 |  |  | 9,031,916 |
| SEQ21 | South East Queensland | Warrill View | 27-Mar-18 |  |  | 11,106,932 |
| SEQ22 | South East Queensland | Warrill View | 27-Mar-18 |  |  | 14,546,030 |
| FCS^a^ | -- | -- | -- |  |  | 13,981,700 |

^a^FCS: Fetal calf serum RNA used as negative control
