# Supplementary material for: Metagenomic Analysis of the Virome of Mosquito Excreta
Source: mSphere. 2020 Sep 9;5(5):e00587-20. doi: 10.1128/mSphere.00587-20 (PMC7485684; doi:10.1128/mSphere.00587-20)
Supplement: TABLE S2 [file mSphere.00587-20-st002.docx]

**Table S2.** Mosquitoes from White Rock (FNQ8) and Cattana Wetlands (FNQ21), far North Queensland, Australia, processed for virus detection from traps that yielded excreta samples positive for Ross River and Barmah Forest viruses.

| **Species** | **No. of pools** | **No. of mosquitoes** | **RRV detections** | **BFV detections** |
| --- | --- | --- | --- | --- |
| *Aedes kochi*^a^ | 5 | 115 | 1 |  |
| *Ae. notoscriptus* | 3 | 85 | 1 |  |
| *Ae. vigilax* | 2 | 23 | 1 |  |
| *Aedes* spp.^b^ | 1 | 3 |  |  |
| *Anopheles annulipes sensu lato*^c^ | 1 | 2 |  |  |
| *An. farauti sensu lato* | 4 | 87 | 1 | 1 |
| *Coquillettidia crassipes* | 3 | 29 |  |  |
| *Culex annulirostris* | 3 | 71 |  | 2 |
| *Cx. gelidus* | 1 | 2 |  |  |
| *Cx. hilli* | 1 | 1 |  |  |
| *Cx. pullus* | 1 | 1 |  |  |
| *Cx. sitiens* | 2 | 51 |  |  |
| *Mansonia septempunctata* | 1 | 2 |  |  |
| *Ma. uniformis* | 1 | 8 |  |  |
| *Tripteroides magnesianus* | 2 | 14 |  |  |
| *Verrallina carmenti* | 6 | 132 |  | 2 |
| *Ve. funerea* | 3 | 46 | 1 |  |
| *Ve. lineata* | 2 | 31 |  |  |
| Total | 42 | 703 | 5 | 5 |

^a^Mosquitoes with profusely spotted wings, femora and tibia belonging to the *kochi* subgroup were classified as *Aedes kochi*.

^b^Mosquitoes belonging to the *Aedes* genus, but unable to be identified to species.

^c^Both *Anopheles annulipes* and *An. farauti* are species complexes, the members of which cannot be reliably distinguished morphologically.
