# Supplementary material for: Metagenomic Analysis of the Virome of Mosquito Excreta
Source: mSphere. 2020 Sep 9;5(5):e00587-20. doi: 10.1128/mSphere.00587-20 (PMC7485684; doi:10.1128/mSphere.00587-20)
Supplement: TABLE S3 [file mSphere.00587-20-st003.docx]

**Table S3.** rRNA contamination in mosquito excreta samples determined by BLAST against SILVA SSU and LSU databases.

| Sample | Source | SSU^a^ | | | LSU^b^ | | |
| --- | --- | --- | --- | --- | --- | --- | --- |
|  |  | hit | no hit | % | hit | no hit | % |
| A | Laboratory infected | 254 | 746 | 25% | 291 | 709 | 29% |
| B | Laboratory infected | 289 | 711 | 29% | 318 | 682 | 32% |
| C | Laboratory infected | 223 | 777 | 22% | 527 | 473 | 53% |
| D | Laboratory infected | 548 | 452 | 55% | 596 | 404 | 60% |
| E | Laboratory infected | 212 | 788 | 21% | 618 | 382 | 62% |
| F | Laboratory infected | 583 | 417 | 58% | 823 | 177 | 82% |
| FNQ1 | Field collected | 589 | 411 | 59% | 718 | 282 | 72% |
| FNQ2 | Field collected | 634 | 366 | 63% | 741 | 249 | 74% |
| FNQ5 | Field collected | 671 | 329 | 67% | 795 | 205 | 80% |
| FNQ6 | Field collected | 718 | 282 | 72% | 867 | 133 | 87% |
| FNQ7 | Field collected | 564 | 436 | 56% | 745 | 255 | 75% |
| FNQ8 | Field collected | 478 | 522 | 48% | 629 | 371 | 63% |
| FNQ9 | Field collected | 478 | 522 | 48% | 690 | 310 | 69% |
| FNQ12 | Field collected | 665 | 335 | 67% | 825 | 175 | 83% |
| FNQ13 | Field collected | 739 | 261 | 74% | 893 | 107 | 89% |
| FNQ14 | Field collected | 678 | 322 | 68% | 801 | 199 | 80% |
| FNQ22 | Field collected | 658 | 342 | 66% | 806 | 194 | 81% |
| FNQ23 | Field collected | 736 | 264 | 74% | 851 | 149 | 85% |
| FNQ24 | Field collected | 745 | 255 | 75% | 931 | 69 | 93% |
| SEQ4 | Field collected | 673 | 327 | 67% | 797 | 203 | 80% |
| SEQ5 | Field collected | 437 | 563 | 44% | 507 | 493 | 51% |
| SEQ6 | Field collected | 123 | 877 | 12% | 192 | 808 | 19% |
| SEQ8 | Field collected | 217 | 783 | 22% | 265 | 735 | 27% |
| SEQ9 | Field collected | 520 | 480 | 52% | 632 | 368 | 63% |
| SEQ13 | Field collected | 497 | 503 | 50% | 588 | 412 | 59% |
| SEQ15 | Field collected | 611 | 389 | 61% | 677 | 323 | 68% |
| SEQ19 | Field collected | 568 | 432 | 57% | 649 | 351 | 65% |
| SEQ20 | Field collected | 637 | 363 | 64% | 758 | 242 | 76% |

^a^Small subunit rRNA

^b^Large subunit rRNA
